# Exploring Substance Abuse and the Dark Tetrad in Health Sciences and Non-Health Sciences Students

**DOI:** 10.3390/bs13090778

**Published:** 2023-09-18

**Authors:** Marina Carvalho de Lima Moraes, Giulia Cunha Russo, Julia da Silva Prado, Ariela Raissa Lima-Costa, Bruno Bonfá-Araujo, Julie Aitken Schermer

**Affiliations:** 1 Department of Psychology, São Francisco University, Campinas 13045-510, Brazil; psicologamarinamoraes@outlook.com (M.C.d.L.M.); giulia.russo@mail.usf.edu.br (G.C.R.); julia.silva.prado@mail.usf.edu.br (J.d.S.P.); arielalima10@gmail.com (A.R.L.-C.); 2 Department of Psychology, University of Western Ontario, London, ON N6A 3K7, Canada; bbonfaar@uwo.ca; 3 Department of Psychology and Management and Organizational Studies, University of Western Ontario, London, ON N6A 3K7, Canada

**Keywords:** alcohol, drugs, Machiavellianism, narcissism, psychopathy, sadism

## Abstract

Substance abuse can be used as a coping strategy to manage stress related to academic activities and is a risk-taking behavior that is also associated with people with higher levels of the Dark Tetrad personality traits. Our study aimed to investigate the association between substance abuse and the Dark Tetrad in students in health and non-health sciences fields. Our sample was composed of 174 college students between 18 and 58 years old (M = 25.60; SD = 9.14). Students completed self-report psychopathy, narcissism, Machiavellianism, sadism, and substance use scales. Results suggest that men consumed more substances and scored higher on the Dark Tetrad than women. Also, when comparing fields, men from health sciences tended to score higher on dark personality traits. These results emphasize the potential risk factors associated with dark personality traits and the consumption of licit and illicit substances by college students, highlighting the need for further studies with this population and the impact of these behaviors and characteristics on future professional practice.

## 1. Introduction

The pressures of a health sciences course can affect students, especially when they are studying away from home, need to maintain their academic performance, and are in touch with people with different medical conditions due to their program requirements [1]. Emotional and psychological stress can externalize itself in a physical or psychological way, such as reduced concentration, memory, and decision-making capacity [2]. Burnout, anxiety, depressive disorders, feelings of fear, incompetence, anguish, anger, and guilt experienced by those dealing with stressors may increase the use of substances to camouflage the emotions and manage the stress [3,4], which might lead to a reduction in empathetic attitudes on the practice [5]. The use of psychoactive substances can also be associated with people who behave in a risky, impulsive, and aggressive manner [6], behaviors that align with the characteristics of the Dark Tetrad of personality [7]. Thus, the aim of this article is to investigate the relationship between psychoactive substance use and the Dark Tetrad and to compare these variables among health sciences and non-health sciences students.

Healthcare students and professionals are subject to a high level of stress. The health field can be considered rigid and demanding. Other factors, such as contact with diseases and death, a predisposition to depressive conditions, and anxious reactions, add to additional possible stressors. Individuals differ as to how they try to suppress the discomfort felt because of stressors, and depending upon their personality and current environment, some may abuse substances [8,9,10]. As is well known, personality traits can influence academic major choices, as well as risky behaviors such as substance abuse. One group of socially undesirable personality traits that can be associated with these outcomes is the Dark Tetrad.

The Dark Tetrad is considered here because of its association with risky behaviors, including substance abuse [11,12]. The tetrad consists of the subclinical forms of psychopathy and narcissism, Machiavellianism, and everyday sadism [13]. Psychopathy characterizes behaviors of callousness and impulsivity (it can be further broken down into primary and secondary psychopathy, with the first one focusing on interpersonal traits such as callousness, and the second focusing on antisocial behavior such as impulsivity); narcissism can be described by self-centered and grandiosity behaviors (it can also be broken down into grandiose and vulnerable narcissism, with the first one focusing on the need for admiration and grandiosity, while the second focusing on insecurity and hypersensitivity to criticism); Machiavellianism is characterized by using manipulative strategies aimed at the exploitation of others and personal gain [7,14], while everyday sadism describes those who extract pleasure in seeing or promoting suffering to others [15]. Although these traits have distinct origins, they have common characteristics, such as manipulative behavior, a grandiose sense of self, and a tendency to exploit others.

### Dark Tetrad and Risky Behaviors

Individuals with high scores on dark aspects of personality tend to have difficulty controlling and identifying their emotions [16]. Common behaviors, including impulsivity (i.e., engaging in risky actions without considering the consequences) and a lack of empathy (i.e., leading to indifference for the well-being of others), underpin the association between the Dark Tetrad and risky behaviors. Studies show positive associations between substance use and psychopathy, narcissism, and sadism [17], although the results with Machiavellianism show mixed results [11,18].

Machiavellians engage in risky behaviors that benefit their self-interest, such as deception, manipulation, and exploitation. This can be shown in interpersonal and professional relationships, where they calculate risks to advance to achieve power [7]. Narcissists display risky behaviors to keep their self-esteem and seek attention, which includes a willingness to take risks that can harm themselves and others. Additionally, they are prone to substance abuse as a means of self-gratification [7,12]. Psychopaths are known for engaging in risky behaviors without experiencing the same levels of anxiety or remorse, leading to illicit activities, substance abuse, and thrill-seeking behaviors. Also, their impulsivity and disregard for outcomes make them more likely to engage in high-risk actions that could harm themselves or others [7,12,17]. Finally, sadists display risky behaviors, such as engaging in violent acts, participating in extreme sports, or pursuing situations to exert control and dominance over others [7,12,15,17].

## 2. Present Study

The Dark Triad was previously found to be higher in economic/business when compared to psychology, law, and political sciences students [19]. When examining the social vocational interest factor, which includes social science, personal service, teaching, social service, and elementary education from the Jackson Career Explorer [20], Kowalski et al. [21] reported nonsignificant correlations with narcissism and significant negative correlations with Machiavellianism and psychopathy. This is the closest vocational interest factor to health science studies. This pattern of correlations was similar to those reported by Jonason et al. [22], who found nonsignificant correlations between social interests using Holland’s [23] model of vocational interests and psychopathy and a negative correlation between Machiavellianism and social interests. Surprisingly, Jonason et al. [22] reported a positive correlation between narcissism and social interests, a result contrary to that reported by Kowalski et al. [21]. In a recent examination of the facets of narcissism and vocational interests, Velji et al. [24] reported that social interests positively correlated with a need for admiration and negatively with a lack of empathy. Each of the above studies examined the Dark Triad (did not include everyday sadism), and there are no studies that we know of that directly relate health sciences students to the components of the Dark Tetrad. Accordingly, the aim of this study is to investigate dark personality traits (i.e., Machiavellianism, narcissism, psychopathy, and everyday sadism) and substance use between health sciences versus non-health sciences students. Because of a lack of previous studies comparing the health and non-health sciences, our study is exploratory, and no hypotheses were established.

## 3. Materials and Methods

### 3.1. Participants and Procedure

We initially conducted a power analysis using G*Power 3.1.9.7 [25] to determine the required sample size for our study. The analysis was performed for a two-tailed *F*-test with an alpha level of 0.05, a power of 0.95, and an effect size (Cohen’s d) of 0.10. The power analysis indicated a minimum sample size of 92 participants.

Our sample was composed of 174 students from the same Brazilian university, aged between 18 and 58 years (M = 25.60; SD = 9.14), 82.75% women, and divided into two groups. Group A comprised undergraduate students in the health sciences field (134 people, 32.83% being undergraduate students in Psychology, 26.86% in Biomedicine, 18.65% in Nursing, 17.16% in Dentistry, 2.98% in Medicine, and 1.49% in Physiotherapy), and group B comprised 40 non-health sciences students; 30.46% were undergoing, at the time of data collection, or had already undergone psychiatric treatment; and 79.3% were taking or had already taken psychiatric medication. Participants were not compensated (i.e., monetarily or by grade) for being in the study. There were no missing data. Further information about the participants can be found in Table 1.

Data collection occurred via the online platform Google Forms, and the link was sent to participants using the university’s online system, which also ensured that they could fill out the form only once. In addition, participants answered a sociodemographic questionnaire and the Informed Consent Form. The link was posted on the social media page of the researchers and the research group (i.e., Facebook and Instagram) and sent via e-mail to coordinators of health sciences courses of a higher education institution, in addition to WhatsApp groups for college students.

### 3.2. Instruments

#### 3.2.1. Levenson Self-Report Psychopathy Scale—LSRP [26]

This scale consisted of 26 self-report items answered using a Likert-type format (1 = Strongly disagree to 5 = Strongly agree). The instrument was composed of two dimensions (i.e., primary psychopathy and secondary psychopathy). Good internal consistency indices were found in our study (α = 0.76 for primary psychopathy and α = 0.71 for secondary psychopathy).

#### 3.2.2. Pathological Narcissism Inventory—PNI [27]

The instrument consisted of 52 self-report items answered in a Likert-type format (0 = Not at all like me to 5 = Very like me). The instrument was composed of two dimensions (i.e., grandiose and vulnerable narcissism). Good internal consistency indices were found in our study (α = 0.91 for grandiose narcissism and α = 0.93 for vulnerable narcissism).

#### 3.2.3. Five Factor Machiavellianism Inventory—FFMI [28]

This instrument consisted of 52 self-report items answered using a Likert-type scale (1 = Strongly disagree to 5 = Strongly agree). The instrument was composed of three factors (i.e., antagonism, agency, and planfulness). Good internal consistency indices were found in our study (α = 0.73 for antagonism, α = 0.78 for agency, and α = 0.79 for planfulness).

#### 3.2.4. Short Sadistic Impulse Scale—SSIS [29]

An instrument that aimed to measure everyday sadism using 10 self-report items. The items were answered in a dichotomous format (0 = Not related to me and 1 = Related to me). Good internal consistency indices were found in our study α = 0.75 for the general scale.

#### 3.2.5. The Alcohol, Smoking, and Substance Involvement Screening Test—ASSIST [30]

This instrument was developed by the WHO and aimed to assess the use of alcohol, tobacco, and other substances via eight subscales and 71 self-report items.

### 3.3. Data Analysis

Initially, Pearson’s correlation analysis was performed to verify the relationship between the study variables. In addition, we performed two multivariate analyses of variance (MANOVA) to investigate the extent to which levels of cannabis, tobacco, hallucinogenic, and alcohol use varied for men and women health sciences students and non-health sciences students. We performed a second MANOVA to investigate the extent to which levels of psychopathy, Machiavellianism, narcissism, and everyday sadism varied for men and women in health sciences students and non-health sciences students. Bootstrapping procedures (1000 resamples; 95% CI) were performed due to the sample difference between the compared groups because this technique allows correction for deviations from the normality of the sample distribution and differences between group sizes and provides a 95% confidence interval for the differences between the means [31]. We employed a bootstrapping procedure involving 1000 resamples, in line with the default settings of the software used (i.e., SPSS), and a common practice in statistical analysis.

## 4. Results

The correlations between the variables are presented in Table 2. To control Type I Error, only those correlations with an alpha of less than 0.001 were deemed to be significant. The correlations show that there are positive relations between secondary psychoactive and the agency subscale of narcissism with using tobacco, alcohol, cannabis, and hallucinogens (with vulnerable narcissism only). Machiavellianism had a negative correlation with using hallucinogens. Everyday sadism showed a positive relationship with alcohol use. A multivariate analysis of variance (MANOVA) was computed to investigate the extent to which levels of cannabis, tobacco, hallucinogens, and alcohol use varied for men and women in health sciences students and non-health sciences students. Table 3 presents the descriptive statistics for all groups.

The BOX’s M test did not meet the assumption of homogeneity of covariance (BOX’S M = 103.700; F (20, 8226.670) = 4.783, *p* < 0.001); therefore, we analyzed Pillai’s trace, which is robust to deviations from multivariate normality and homogeneity of covariance of the matrices. The MANOVA results showed that there was no main effect for sex (F (4, 166) = 0.995, *p* = 0.412; η^2^ = 0.023), nor for the sex*field interaction (F (4, 166) = 0.475, *p* = 0.754; η^2^ = 0.011). Only the field of study variable showed statistically significant results but with low effect sizes (F (4, 166) = 2.733; *p* = 0.031; η^2^ = 0.062). A posteriori tests (Bonferroni post hoc) showed that, in relation to health sciences and non-health sciences students, only the variable hallucinogens showed statistically significant differences, with non-health sciences students showing a higher level of consumption than health sciences students (*p* = 0.02; 95% CI = −0.86, −0.07; d = 0.49).

A second multivariate analysis of variance (MANOVA) was calculated to investigate the extent to which levels of psychopathy, Machiavellianism, narcissism, and everyday sadism varied for men and women in health sciences students and non-health sciences students. Descriptive statistics for all groups are presented as Supplementary Material. The BOX’s M test did not meet the assumption of homogeneity of covariance [BOX’S M = 207.041; F (108, 6210.394) = 1.550; *p* < 0.001]; therefore, we analyzed from Pillai’s trace. MANOVA results showed that there was no main effect for the field [F (8, 163) = 1.931, *p* = 0.059; η^2^ = 0.087]. Statistically significant results were found in the main effect for sex [F (8, 163) = 3.594, *p* = 0.001; η^2^ = 0.150] and for the sex*field interaction [F (8, 163) = 2.661, *p* = 0.009; η^2^ = 0.116]. A posteriori tests (Bonferroni post hoc) showed that for men and women, a significant difference was found for primary psychopathy (*p* = 0.01; 95% CI = 1.607, 6.533; d = 5.64), antagonism (*p* = 0.02; 95% CI = 4.942, 12.694; d = 7.82), planfulness (*p* = 0.02; 95% CI = 0.220, 7.529; d = 3.64); with men showing higher levels (primary psychopathy: M = 33.11, SD = 1.08; antagonism: M = 54.50, SD = 1.70; planfulness: M = 44.51, SD = 1.60) than women (primary psychopathy: M = 29.04, SD = 0.63; antagonism: M = 45.68, SD = 0.98; planfulness: M = 40.63, SD = 0.93).

Regarding the interaction between the field of study and self-report biological sex, a posteriori tests (i.e., post hoc Bonferroni) showed significant differences for primary psychopathy, antagonism, and agency. In relation to primary psychopathy, men from the health sciences field (M = 33.76, SD = 1.42) showed higher levels than women from the health sciences field (M = 29.53, SD = 0.54; *p* = 0.01; 95% CI = 1.23, 7.24; d = 6.02). Regarding antagonism, men from the health sciences field (M = 55.00, SD = 2.24) showed higher levels than women from health sciences field (M = 45.18, SD = 0.85; *p* < 0.01; 95% CI = 5.09, 14.55; d = 8.87); and men from non-health sciences fields (M = 54.00, SD = 2.56) had higher levels than women from non-health sciences fields (M = 46.18, SD = 1.78; *p* = 0.01; 95% CI = 1.67, 13.96; d = 3.9).

In relation to the agency trait, men from the health sciences field (M = 68.06, SD = 2.75) showed higher levels than women from the health sciences field (M = 60.33, SD = 1.05; *p* = 0.01; 95% CI = 1.23, 7.24; d = 5.67) and then men from non-health sciences field (M = 56.92, SD = 3.15; *p* = 0.01; 95% CI = 2.88, 19.39; d = 3.94). Women from non-health sciences fields (M = 65.30, SD = 2.18) had higher levels than men from non-health sciences fields (M = 59.92, SD = 3.15; *p* = 0.03; 95% CI = −15.94, −0.81; d = 2.18) and then women from health sciences field (M = 60.33, SD = 1.05; *p* = 0.04; 95% CI = −9.75, −0.18; d = 3.76).

## 5. Discussion

The aim of this article was to investigate the Dark Tetrad traits and substance use among health sciences students and non-health sciences students. Our study has two main results. First, only hallucinogens showed a significant difference between the groups, with non-health sciences students having higher levels of substance use when compared to health sciences students. Second, in general, men from health sciences had higher results of socially undesirable traits.

The results of our study reinforce the evidence that people with higher scores in the Dark Tetrad tend to exhibit more risky behaviors, including substance use [18]. A positive relationship was found between drug use, especially tobacco, alcohol, cannabis, and hallucinogens, which are more frequently used substances; the exception was the agency and planfulness dimensions of Machiavellianism. Although the correlations were weak to moderate, they demonstrate a pattern in the engagement of risky behavior, a common component of the tetrad traits in line with previous studies (e.g., [18,32]).

Trait-specific characteristics may explain the positive associations we found. Considering the psychopathy dimensions, secondary psychopathy was more related to legal and illegal psychoactive drugs (i.e., tobacco, alcohol, cannabis, and hallucinogens), which is consistent with this personality trait, which is characterized by antisocial behavior, impulsivity, and anxiety [33]. For narcissism, vulnerable and grandiose dimensions showed similar magnitudes, with vulnerable narcissism also showing a positive relationship with hallucinogens. One of the aspects that can explain this relationship is that people with high levels of grandiose narcissism tend to have a greater perception of superiority and need to show off and consume psychoactive drugs to pass an image of someone unbeatable, focusing only on the short-term gain of drug use [34], while people with high levels of vulnerable narcissism make use of psychoactive drugs as a way to escape feelings of inferiority and shame, thus using drugs help them to fit in and be accepted by others [35]. In contrast, Machiavellianism (i.e., agency and planfulness) showed a tendency toward negative relations with psychoactive substance use. People with high levels of Machiavellianism tend to focus more on maintaining power and control [36], which may be impaired by substance use. Finally, tobacco and alcohol use by people with higher levels of everyday sadism may be related more to recreational use and risky behavior associated with this personality trait [15].

Considering the context of the field of study, our results indicate that only the consumption of hallucinogens differs between health sciences students and non-health sciences students, with the latter tending to consume more. The use of hallucinogens is strongly associated with the consumption of other drugs, such as cannabis, inhalants, and sedatives. The prevalence of hallucinogen use among Brazilian undergraduates is estimated at approximately 7.6%. When broken down by gender, this figure rises to 11% among men and slightly lower at 4.9% among women. Moreover, when considering the type of university attended, students from private institutions exhibit a higher prevalence (8.5%) when compared to their counterparts from public institutions (4.3%). An examination of academic fields reveals a divergence among students majoring in natural sciences (8.9%), social sciences (7.8%), and health sciences (5.1% [37]). The long-term or therapeutic effects of these drugs are still unclear [38]. Thus, it is necessary to further investigate what motivates college students to consume drugs, especially hallucinogens.

When comparing the Dark Tetrad levels between health sciences students and non-health sciences students, we noticed a tendency for male health science students to present higher levels of aversive traits. Possibly, this occurs due to a cumulative and cyclical effect since men already tend to present higher levels of the Dark Tetrad traits [39] and to be more aggressive [40]. Also, substance use was shown to be related to a decrease in academic performance, especially in the health context [41].

## 6. Conclusions

Some limitations should be considered. First, our sample of students from different science fields was small, which can limit the generalizability of our findings. It should also be noted that we only investigated each field for health sciences; thus, non-health science participants were not required to answer about their major. However, our goal was to compare general fields, and we were able to achieve that. Second, our sample was mostly composed of women. This sex imbalance can limit the generalization of our results, so we suggest future studies seek a more balanced sample. Third, we focused on the Dark Tetrad and substance use. However, other relevant variables, such as socio-economic status or cultural factors, were not included in the analysis. Thus, future studies could try to identify if substance use is influenced by culture or status. Despite the limitations mentioned, our study has several positive aspects that contribute to its strength. Our findings contribute to the understanding of the complex relationships between personality traits and substance use among college students. The results highlight the need for targeted interventions and preventive measures to address the potential risk factors associated with dark personality traits and psychoactive substance use. Future research can explore additional factors and examine longitudinal relationships to provide further evidence of the underlying mechanisms and potential implications for intervention strategies.

## Figures and Tables

**Table 1:** Sociodemographic information.

		N	%
Civil status	Single	139	79.9
	Married	27	15.5
	Divorced	8	4.6
Region	South	4	2.3
	Southeast	159	91.4
	Midwest	7	4
	North	4	2.3
City size	Small-sized cities or rural areas (less than 100 thousand inhabitants)	61	35.1
	Medium-sized cities (between 100 and 500 thousand inhabitants)	83	47.7
	Large-sized cities (more than 500 thousand inhabitants)	27	15.5
	Capital	3	1.7
Religion	No religion	55	31.6
	Catholic (i.e., Apostolic, Roman, Orthodox)	63	36.2
	Evangelicals (i.e., Mission, Pentecostal, others)	27	15.5
	Spiritist	18	10.3
	Afro-Brazilian religion (i.e., Umbanda)	10	5.7
	Buddhists	1	0.6
Employment status	Public employees	7	4
	Private employees	45	25.9
	Self-employed	30	17.2
	Unemployed	77	44.3
	Students with a research scholarship	11	6.3
	Retired	4	2.3

**Table 2:** Correlation between personality traits and psychoactive use.

	1	2	3	4	5	6	7	8	9	10	11	12	13	14	15	16
1. Primary psychopathy	1															
2. Secondary psychopathy	0.31 *	1														
3. Vulnerable narcissism	0.04	0.53 *	1													
4. Grandiose narcissism	0.35 *	0.59 *	0.71 *	1												
5. Antagonism (Machiavellianism)	0.62 *	0.31 *	−0.01	0.34 *	1											
6. Agency (Machiavellianism)	0.18	−0.29 *	−0.36 *	−0.05	0.16	1										
7. Planfulness (Machiavellianism)	−0.04	−0.57 *	−0.22 *	−0.17	−0.06	0.30 *	1									
8. Sadism	0.59 *	0.37 *	0.12	0.42 *	0.47 *	0.13	−0.26 *	1								
9. Tobacco	0.19	0.33 *	0.29 *	0.34 *	0.13	−0.03	−0.20 *	0.18	1							
10. Alcohol	0.17	0.36 *	0.26 *	0.32 *	0.16	−0.01	−0.20 *	0.22 *	0.59 *	1						
11. Cannabis	0.08	0.27 *	0.31 *	0.26 *	0.07	−0.08	−0.18	0.06	0.62 *	0.52 *	1					
12. Cocaine	0.03	0.01	0.07	0.01	0.05	−0.09	−0.04	0.11	0.13	0.18 *	0.28 *	1				
13. Amphetamine	0.01	0.06	0.02	0.02	0.03	−0.08	−0.13	−0.04	0.20 *	0.22 *	0.27 *	0.27 *	1			
14. Inhalants	0.01	0.11	0.15	0.09	0.06	−0.18	−0.12	−0.01	0.32 *	0.38	0.30 *	0.52 *	0.40 *	1		
15. Sedatives	0.07	0.10	0.16	0.16	0.07	−0.19	−0.09	0.18	0.14	0.15	0.10	0.25 *	0.34 *	0.27 *	1	
16. Hallucinogens	0.01	0.21 *	0.23 *	0.10	0.04	−0.25 *	−0.12	−0.03	0.29 *	0.23 *	0.46 *	0.27 *	0.42 *	0.51 *	0.32 *	1
17. Opioids	0.07	0.02	0.12	0.08	0.01	−0.11	−0.08	0.10	0.07	0.07	0.08	0.25 *	0.13	0.18	0.45 *	0.14

* *p* < 0.001.

**Table 3:** Descriptive statistics for the variables substance type subdivided by sex and field.

			Mean	SD	N
Cannabis	Men	Health Sciences	4.24	1.52	17
		Non-Health Sciences	5.08	0.28	13
		Total	4.60	1.22	30
	Women	Health Sciences	5.22	2.17	117
		Non-Health Sciences	5.35	1.65	26
		Total	5.24	2.08	143
	Total	Health Sciences	5.10	2.12	134
		Non-Health Sciences	5.26	1.35	39
		Total	5.13	1.97	173
Tobacco	Men	Health Sciences	5.53	2.53	17
		Non-Health Sciences	4.92	0.86	13
		Total	5.27	1.98	30
	Women	Health Sciences	5.63	2.51	117
		Non-Health Sciences	5.31	1.19	26
		Total	5.57	2.32	143
	Total	Health Sciences	5.62	2.50	134
		Non-Health Sciences	5.18	1.10	39
		Total	5.52	2.27	173
Hallucinogens	Men	Health Sciences	4.53	0.80	17
		Non-Health Sciences	5.00	0.00	13
		Total	4.73	0.64	30
	Women	Health Sciences	4.62	1.07	117
		Non-Health Sciences	5.08	0.39	26
		Total	4.70	0.99	143
	Total	Health Sciences	4.60	1.03	134
		Non-Health Sciences	5.05	0.32	39
		Total	4.71	0.94	173
Alcohol	Men	Health Sciences	6.18	1.85	17
		Non-Health Sciences	6.77	2.80	13
		Total	6.43	2.28	30
	Women	Health Sciences	7.28	3.18	117
		Non-Health Sciences	7.50	2.94	26
		Total	7.32	3.13	143
	Total	Health Sciences	7.14	3.06	134
		Non-Health Sciences	7.26	2.88	39
		Total	7.17	3.01	173

## Data Availability

Data are available per reasonable request from the corresponding author.

## Supplementary Materials

The following supporting information can be downloaded at https://osf.io/rtnve/?view_only=899f39209e4846e493d97ff1d6ff9d12 (accessed on 16 September 2023), Table S1: Descriptive statistics of psychopathy, Machiavellianism, sadism and narcissism by sex and field.
